# Survival outcomes of intensity-modulated radiotherapy combined with immunotherapy and targeted therapy for hepatocellular carcinoma with classification I–IV portal vein tumor thrombus: a propensity score matching study

**DOI:** 10.3389/fimmu.2026.1775165

**Published:** 2026-07-28

**Authors:** Lijun Chen, Chunmi Wei, Zhehao Xiao, Qiaoyuan Wu, Liqing Li, Bangde Xiang, Shichun Guan, Peng Zhao, Shixiong Liang, Jianxu Li

**Affiliations:** 1 Department of Radiation Oncology, Guangxi Medical University Cancer Hospital, Nanning, China; 2 Department of Hepatobiliary Surgery, Guangxi Medical University Cancer Hospital, Nanning, China; 3 Department of Orthopedic Surgery, The First People’s Hospital of Xiangtan City, Xiangtan, Hunan, China; 4 The People’s Hospital of Chongzuo, Youjiang Medical University for Nationalities, Chongzuo, China

**Keywords:** hepatocellular carcinoma, immunotherapy, portal vein tumor thrombus, radiotherapy, targeted therapy

## Abstract

**Purpose:**

The study aimed to evaluate the efficacy and safety of intensity-modulated radiotherapy (IMRT) combined with immunotherapy and targeted therapy (IT) in hepatocellular carcinoma (HCC) patients with portal vein tumor thrombus (PVTT) classification I–IV.

**Patients and methods:**

The study analyzed HCC patients with PVTT who received IMRT combined with immunotherapy and targeted therapy (RT+IT, *n* = 176) or immunotherapy and targeted therapy alone (IT, *n* = 136). Propensity score matching (PSM) was performed to reduce baseline imbalance. Comparisons were made between the two groups in overall survival (OS), progression-free survival (PFS), and treatment-related adverse events. Subgroup analyses were further conducted to evaluate OS and PFS across PVTT classification I–IV. Cox regression analysis was performed to identify independent prognostic factors in patients receiving RT+IT.

**Results:**

Following PSM, 97 pairs of participants were matched. In RT+IT, the median OS was longer (27.63 months, 95% CI: 20.00–33.00) compared to IT (17.57 months, 95% CI: 15.20–22.10), with a hazard ratio (HR) of 0.467 (95% CI: 0.323–0.675, *p* < 0.001). The cumulative OS rates at 3, 6, 12, 24, and 36 months were 99.0%, 96.9%, 83.3%, 54.2%, and 27.6% for RT+IT vs. 99.0%, 91.6%, 76.8%, 27.1%, and 12.2% for IT (*p* < 0.001). RT+IT had a longer median PFS (12.30 months,95% CI: 10.47–16.45) than IT (7.20 months, 95% CI: 6.37–9.67) (*p* < 0.001). Subgroup analysis by PVTT classification revealed that RT+IT was associated with improved OS and PFS across PVTT I, II, III, and IV than IT. Grade 3–4 adverse events between the two groups had no significant statistical difference. Biologically effective dose (BED10, *α*/*β* = 10 Gy), alpha fetoprotein (AFP), Child–Pugh (CP) grade, Cheng’s classification of PVTT, and combined transcatheter chemoembolization (TACE) were identified as independent predictors of OS in patients who underwent RT+IT.

**Conclusion:**

IMRT combined with immunotherapy and targeted therapy was associated with improved survival outcomes with a manageable safety profile in HCC patients with PVTT across classification I–IV.

## Introduction

With over 830,000 deaths and 906,000 new cases every year, according to the GLOBOCAN 2020 database, primary liver cancer (8.3%) ranks third globally in terms of cancer-related deaths, and 75%–85% of these patients have hepatocellular carcinoma (HCC) (1). When HCC is first diagnosed, between 10% and 40% of patients have portal vein tumor thrombus (PVTT) (2), which is classified as advanced stage C based on the Barcelona Clinic Liver Cancer (BCLC) staging system (3).

The median overall survival (OS) for HCC patients with PVTT is 2–4 months if treatment is not received (4, 5). The National Comprehensive Cancer Network (NCCN) guidelines recommend immunotherapy plus targeted therapy (atezolizumab plus bevacizumab) as the first-line therapy for advanced HCC (6). However, this combination therapy offers an objective response rate (ORR) of approximately 30% and a median progression-free survival (PFS) of only 6.8 months, which is suboptimal (7–9). Radiotherapy (RT) is an important treatment approach when it comes to the multidisciplinary therapy regimen of advanced HCC, especially those with PVTT. Li et al. elucidated that intensity-modulated radiotherapy (IMRT) plus transarterial chemoembolization (TACE) can extend the median OS of HCC patients with PVTT to 10.0 months (10). Moreover, Sang et al. demonstrated that in HCC patients with PVTT treated with RT combined with TACE, the median OS (mOS) was 10.6 months, with 1- and 2-year OS rates of 42.5% and 22.8%, respectively. The 1- and 2-year progression rates for PVTT were 22.1% and 33.3%, respectively. Two to 3 months after completion of RT, the PVTT progression-free rate was 85.6%, with progression-free response defined as complete response, partial response, or stable disease (11). Based on these findings, the Chinese Expert Consensus (2018 Edition) and the Korean Practice Guidelines (2022 Edition) recommend RT as the treatment option for liver tumors unresectable with PVTT (12, 13).

RT combined with immunotherapy theoretically has the effect of synergistic enhancement and synergistic interaction (14). The immunosuppressive state caused by RT can be disrupted by programmed cell death protein 1 (PD-1) inhibitors (15, 16). A retrospective study conducted by Li et al. showed that for patients with advanced HCC, IMRT combined with anti-PD-1 immunotherapy improved survival and exhibited fewer severe toxic effects compared to traditional therapy with TACE plus sorafenib, and the median OS was significantly longer in the RT + anti-PD-1 group (17.4 months) compared to the TACE + sorafenib group (11.9 months). Additionally, grade 3 or higher treatment-related adverse events occurred significantly less frequently in the RT + anti-PD-1 group than in the TACE + sorafenib group (29.7% vs. 75.6%, *p* < 0.001) (17). In patients with advanced HCC, RT combined with immune checkpoint inhibitors (ICIs) and targeted therapy enhanced OS, PFS, and disease control rate (DCR). The median OS was not reached in the RT group, while it was 9.7 months in the ICI + targeted therapy group (*p* = 0.002); the median PFS (mPFS) was 8.3 months in the RT group, compared to 4.2 months in the ICI + targeted therapy group (*p* < 0.001); and the DCR was 100% in the RT group, vs. 75.9% in the ICI + targeted therapy group (*p* = 0.005) (18).

Recent studies have begun to support the role of RT combined with immunotherapy and targeted therapy in HCC with PVTT. Ma et al. reported that adding RT to tyrosine kinase inhibitor (TKI) plus PD-1 inhibitor significantly improved OS and PFS in HCC patients with PVTT (19). In addition, a prospective single-arm phase II trial by Mo et al. showed promising efficacy and manageable toxicity for stereotactic body radiotherapy (SBRT) combined with cadonilimab and lenvatinib in patients with Vp3/Vp4 PVTT, with a median PFS of 6.8 months and a median OS of 13.4 months (20). Although these studies support the potential value of integrating RT into systemic therapy, important gaps remain. The currently available evidence is still limited by a relatively small sample size, restriction to selected PVTT subgroups, and limited data specifically focused on IMRT-based combination strategies across the full spectrum of PVTT classifications. Therefore, the present study aimed to evaluate the efficacy and safety of IMRT combined with immunotherapy and targeted therapy in HCC patients with PVTT classification I–IV.

## Methods

### Patients

This study included 312 HCC patients having PVTT who underwent IMRT at Guangxi Medical University Cancer Hospital between September 2018 and May 2024. The Declaration of Helsinki was followed in the execution of this study plan. The Ethics Committee of Guangxi Medical University Cancer Hospital approved this retrospective study (Ethics No. KY2024873) and dropped the informed consent requirement since the patients could not be recognized.

The primary inclusion criteria were as follows: (1) diagnosis with HCC, confirmed either by histopathology or according to clinical imaging diagnostic standards specified in the Clinical Practice Guidelines (21); 2) diagnosis of intrahepatic HCC with PVTT according to CT or MRI; 3) inclusion in the combination therapy group receiving RT with immunotherapy and targeted therapy (IT), administered concurrently or sequentially, with an interval not exceeding 4 weeks between RT and immunotherapy with targeted therapy; 4) Eastern Cooperative Oncology Group performance score (ECOG PS) of 0–1; 5) Child–Pugh (CP) grade A or B liver function before RT; and 6) RT started no more than 4 weeks after TACE when combined with TACE. The exclusion criteria were 1) CP grade C liver function; 2) ECOG PS ≥2; 3) combined extrahepatic distant metastases; 4) combined intrahepatic cholangiocarcinoma or other malignant tumors; 5) combined severe cardiac, cerebral, pulmonary, renal, and other medical diseases; and 6) missing clinical or follow-up data. Ultimately, the study included 312 patients, with 176 receiving RT+IT and 136 receiving IT alone.

### Classification of PVTT

Macroscopic PVTT was divided into four categories based on Cheng’s classification (22). PVTT invasion in the segmental or sectoral branches of the portal vein or above is referred to as classification I, PVTT invasion in the left or right branch of the portal vein is referred to as classification II, PVTT invasion of the main portal vein is referred to as classification III, and PVTT invasion into the superior mesenteric vein is referred to as classification IV.

### Radiotherapy technique

For RT planning, contrast-enhanced CT was performed at 2.5–5 mm slice thickness, with the patient placed in supine position and breathing spontaneously. A 4- to 5-mm margin was placed outside the gross tumor volume (GTV) in order to calculate the clinical target volume (CTV). The size of the tumor thrombus or tumor elevated on contrast-enhanced CT and CT-MRI imaging fusion was encoded into GTV. The CTV and the 5–10-mm buffer were utilized to define the planned target volume to minimize positioning uncertainty and the impact of breathing movement. The Pinnacle3 system (Philips, Netherlands) or the MIM 6.8 system (MIM, USA) was used to delineate all the target areas and organs at risk (OARs). All patients received RT with a 6-MV X-ray machine using a linear accelerator. A dose–volume histogram (DVH) analysis was conducted to evaluate the radiation plan, ensuring the OARs were adequately protected.

The total dose to GTV ranged from 30 to 66 Gy, with 2 to 5 Gy for fraction size. The patients were given a median fraction size of 3.0 Gy (3.0, 3.3 Gy) and a median total dose of 51.0 Gy (50.0, 60.0 Gy). RT was administered over five consecutive days each week.

### Immunotherapy and targeted therapy

The treatment regimen of immunotherapy and targeted therapy drugs was determined empirically by the physician. Patients received IMRT concurrently with or sequentially to ICIs and targeted therapy (the time intervals not exceeding 4 weeks). The administration technique, dosage, and duration adhered to the manufacturer’s recommendations. ICIs included camrelizumab, tislelizumab, sintilimab, and atezolizumab. Antivascular endothelial growth factor antibodies (bevacizumab) and multikinase inhibitors (lenvatinib, apatinib, and sorafenib) were part of the targeted therapy.

### Patients’ follow-up

Before RT, 1 month follow-up, and for the first 2 years, every 2 to 3 months, and every 4 to 6 months for 3 to 5 years, patients were examined using laboratory testing and MRI or contrast-enhanced CT imaging to assess tumor response. The OS was determined by the period of time from beginning of RT to death date for any cause, otherwise to the date of last follow-up. PFS was defined as the interval between the start of IMRT and the disease’s progression, the last follow-up, or death from any cause. The Common Terminology Criteria for Adverse Events (CTCAE) version 5.0 was employed to evaluate adverse events related to treatment.

### Statistical analysis

A 1:1 optimal propensity score matching (PSM) approach was applied to patients in the two groups to reduce between-group heterogeneity and selection bias, using the “MatchIt” package in R software. The *p*-value and standardized mean difference (SMD) value were used to evaluate the balance of the baseline between the two groups after PSM. Quantitative variables were evaluated for normality of distribution using histograms and the Shapiro–Wilk test. The Mann–Whitney *U* test compared quantitative variables with abnormal distributions. The chi-square test compared categorical variables, as well as the Fisher’s exact test. Cox regression analysis selected independent factors affecting OS (*p* < 0.05), and the proportional hazards (PH) assumption for the Cox regression was assessed using time-dependent covariates generated by including interaction terms between each predictor and follow-up time. The log-rank test and the Kaplan–Meier method were adopted to compare OS between groups. The statistical analysis software used was SPSS version 26.0 and R version 4.3.2.

## Results

### Patient characteristics

A total of 312 HCC patients with PVTT, 176 in the RT+IT group and 136 in the IT group, were included in the study. Among the 176 patients who received combination therapy, 120 patients (68.18%) underwent IMRT concurrently with immunotherapy and targeted therapy. In this concurrent-treatment group, immunotherapy was initiated on the same day as IMRT and continued as scheduled throughout the IMRT course. Of these 120 patients, 26 had marked esophagogastric varices on gastroscopy and were considered to be at high risk for gastrointestinal bleeding; therefore, targeted therapy was postponed and initiated only after completion of IMRT. The remaining 56 patients (31.82%) received immunotherapy plus targeted therapy sequentially after completion of IMRT. In the overall cohort of 176 patients, immunotherapy was delayed in 15 patients (8.52%) because of hepatic toxicity, hematologic toxicity, hemorrhagic risk, gastrointestinal symptoms, or poor general condition, whereas targeted therapy was delayed in 35 patients (19.89%). All patients completed at least two scheduled cycles of immunotherapy combined with targeted therapy.

**Table 1** summarizes baseline data before and after PSM, including liver function, clinical aspects, and Cheng’s classification of PVTT. Before PSM, 66 (37.50%) patients received RT + camrelizumab + lenvatinib/apatinib/sorafenib, 82 (46.60%) patients received RT + tislelizumab + lenvatinib/apatinib/sorafenib, 19 (10.80%) patients received RT + sintilimab + lenvatinib/bevacizumab, and 9 (5.10%) patients received RT + atezolizumab + bevacizumab in the RT+IT group. The distribution of PVTT classification (I, II, III, IV) was 28 (15.91%), 72 (40.91%), 59 (33.52%), and 17 (9.66%) in the RT+IT group, compared to 26 (19.12%), 62 (45.59), 36 (26.47), and 12 (8.82) in the IT group. Patients who underwent RT+IT exhibited fewer tumor number (*p* < 0.001), smaller single max tumor size (*p* < 0.001), lower alpha fetoprotein level (*p* = 0.003), and lower AST level (*p* < 0.001). After PSM, 97 patients received RT+IT and 97 underwent IT. The classification of PVTT (I, II, III, IV) was 14 (14.43%), 42 (43.30%), 32 (32.99%), and 9 (9.28%) in the RT+IT group, compared to 15 (15.46%), 39 (40.21%), 32 (32.99%), and 11 (11.34%) in the IT group. Baseline characteristics were balanced between groups (*p* > 0.05, the absolute value of SMD < 0.1).

**Table 1:** Baseline characteristics of HCC patients with PVTT.

Variable	Before PSM				After PSM			
	RT+IT (*n* = 176)	IT (*n* = 136)	*p*	SMD	RT+IT (*n* = 97)	IT (*n* = 97)	*p*	SMD
Age (years), mean ± SD	50.93 ± 10.37	50.57 ± 11.83	0.772	−0.031	51.46 ± 10.50	51.41 ± 12.21	0.975	−0.004
Sex, *n* (%)			0.087				0.420	
Female	12 (6.82)	17 (12.50)		0.172	9 (9.28)	6 (6.19)		−0.098
Male	164 (93.18)	119 (87.50)		−0.172	88 (90.72)	91 (93.81)		0.098
Hepatitis B virus infection, *n* (%)			0.010				0.848	
Negative	47 (26.70)	20 (14.71)		−0.339	16 (16.49)	17 (17.53)		0.027
Positive	129 (73.30)	116 (85.29)		0.339	81 (83.51)	80 (82.47)		−0.027
Radiographic cirrhosis, *n* (%)			0.080				0.884	
Absent	68 (38.64)	66 (48.53)		0.198	41 (42.27)	40 (41.24)		−0.021
Present	108 (61.36)	70 (51.47)		−0.198	56 (57.73)	57 (58.76)		0.021
ECOG PS, *n* (%)			0.627				0.315	
0	87 (49.43)	71 (52.21)		0.056	45 (46.39)	52 (53.61)		0.095
1	89 (50.57)	65 (47.79)		−0.056	52 (53.61)	45 (46.39)		−0.095
Tumor number, *n* (%)			<0.001				0.743	
≤3	79 (44.89)	28 (20.59)		−0.601	26 (26.80)	24 (24.74)		−0.048
>3	97 (55.11)	108 (79.41)		0.601	71 (73.20)	73 (75.26)		0.048
Max tumor size (cm), mean ± SD	8.82 ± 4.02	10.98 ± 4.83	<0.001	0.448	9.64 ± 4.29	10.00 ± 4.60	0.568	0.079
Alpha fetoprotein (ng/mL), *n* (%)			0.003				0.666	
<400	107 (60.80)	60 (44.12)		−0.336	52 (53.61)	49 (50.52)		−0.062
≥400	69 (39.20)	76 (55.88)		0.336	45 (46.39)	48 (49.48)		0.062
Child–Pugh class, *n* (%)			0.088				0.351	
A	132 (75.00)	90 (66.18)		−0.187	70 (72.16)	64 (65.98)		−0.091
B	44 (25.00)	46 (33.82)		0.187	27 (27.84)	33 (34.02)		0.091
ALBI grade, *n* (%)			0.078				0.557	
1	21 (11.93)	26 (19.12)		0.183	17 (17.53)	14 (14.43)		−0.088
2	155 (88.07)	110 (80.88)		−0.183	80 (82.47)	83 (85.57)		0.088
Aspartate aminotransferase (U/L), M (Q_1_, Q_3_)	52.50 (39.75, 83.25)	70.50 (48.75, 115.25)	<0.001	0.165	61.00 (38.00, 94.00)	68.00 (47.00, 109.00)	0.116	0.030
Alanine aminotransferase (U/L), M (Q_1_, Q_3_)	37.50 (24.00, 58.00)	43.00 (28.00, 67.25)	0.187	−0.026	41.00 (24.00, 61.00)	43.00 (27.00, 60.00)	0.847	−0.055
Alkaline phosphatase (U/L), M (Q_1_, Q_3_)	143.50 (106.00, 205.25)	146.50 (112.00, 198.25)	0.432	0.145	158.00 (111.00, 229.00)	143.00 (110.00, 196.00)	0.360	0.060
Prothrombin time (s), mean ± SD	12.93 ± 1.50	12.07 ± 2.02	<0.001	−0.423	12.66 ± 1.38	12.55 ± 1.96	0.654	−0.056
Cheng’s classification of PVTT, *n* (%)			0.540				0.951	
I	28 (15.91)	26 (19.12)		0.082	14 (14.43)	15 (15.46)		0.029
II	72 (40.91)	62 (45.59)		0.094	42 (43.30)	39 (40.21)		−0.063
III	59 (33.52)	36 (26.47)		−0.160	32 (32.99)	32 (32.99)		0.000
IV	17 (9.66)	12 (8.82)		−0.029	9 (9.28)	11 (11.34)		0.065
Combined TACE, *n* (%)			0.792				0.758	
No	43 (24.43)	35 (25.74)		0.030	30 (30.93)	32 (32.99)		0.044
Yes	133 (75.57)	101 (74.26)		−0.030	67 (69.07)	65 (67.01)		−0.044

Values are presented as *n* (%) and interquartile ranges or mean. *p*-values were calculated using a two-sided chi-square test and Mann−Whitney *U* test or Student’s *t*-test.

ALBI, albumin–bilirubin; ECOG PS, Eastern Cooperative Oncology Group-performance status; IT, immunotherapy and targeted therapy; PSM, propensity score matching; PVTT, portal vein tumor thrombus; RT, radiotherapy; SMD, standardized mean difference; TACE, transcatheter chemoembolization; M, median; Q₁, 1st quartile; Q₃, 3rd quartile; SD, standard deviation.

### Overall survival analyses

**Figure 1:**
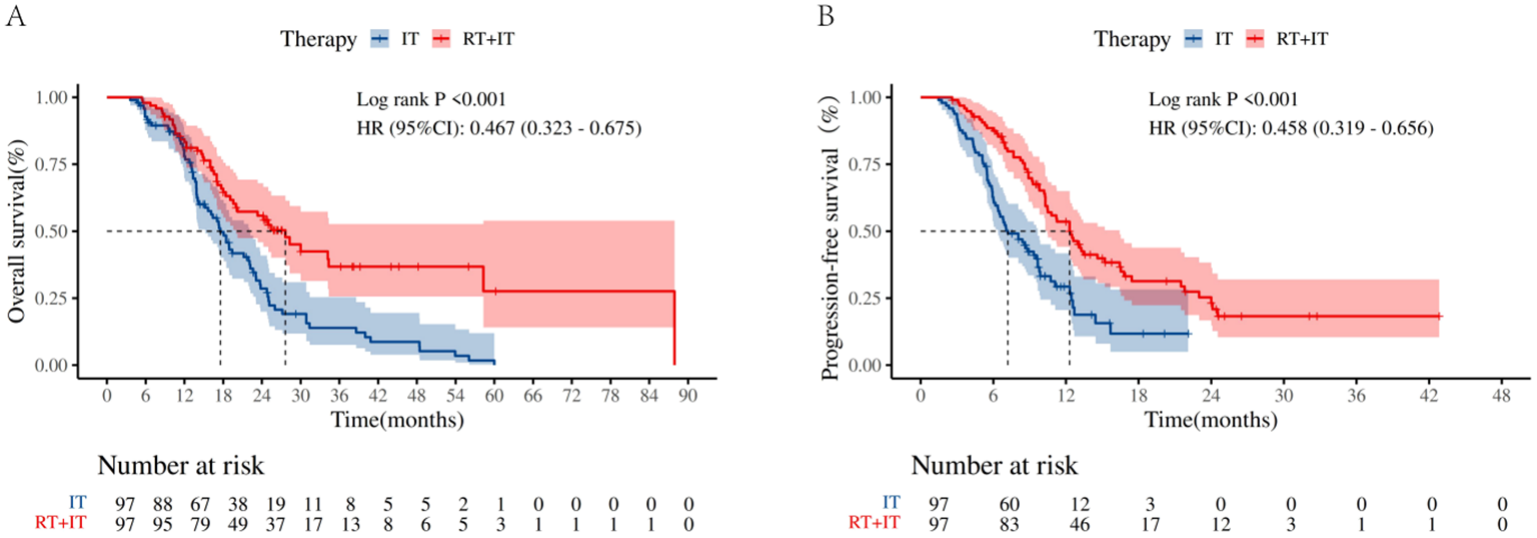
Kaplan–Meier survival curves comparing **(A)** OS (median OS: 27.63 vs. 17.57 months) and **(B)** PFS (median PFS: 12.30 vs. 7.20 months) among patients who underwent RT+IT vs. IT after PSM. PFS, progression-free survival; OS, overall survival; PSM, propensity score matching; RT, radiotherapy; IT, immunotherapy and targeted therapy.

The overall median follow-up duration was 28.30 months [95% confidence interval (CI), 26.30–36.30] for all 312 patients. After PSM, 194 patients were included (97 in the RT+IT group and 97 in the IT group). For OS, there were 122 events in total, including 47/97 in the RT+IT group and 75/97 in the IT group. By PVTT subgroup, the OS events were 7/14 vs. 13/15 for PVTT I, 19/42 vs. 32/39 for PVTT II, 17/32 vs. 22/32 for PVTT III, and 4/9 vs. 8/11 for PVTT IV (RT+IT vs. IT). HCC patients who underwent RT+IT had an mOS of 27.63 months (95% CI: 20.00–33) as opposed to 17.57 months (95% CI: 15.20–22.10) (HR: 0.467, 95% CI: 0.323–0.675, *p* < 0.001) as compared to those who had IT (**Figure 1A**). At 3, 6, 12, 24, and 36 months, the cumulative OS rates of the RT+IT group were 99.0%, 96.9%, 83.3%, 54.2%, and 27.6%, while the cumulative OS rates of the IT group were 99.0%, 91.6%, 76.8%, 27.1%, and 12.2% (*p* < 0.001). Cases with PVTT I–IV in the RT+IT group showed significantly longer OS than the IT group, according to the subgroup analysis of PVTT classification.

**Figure 2:**
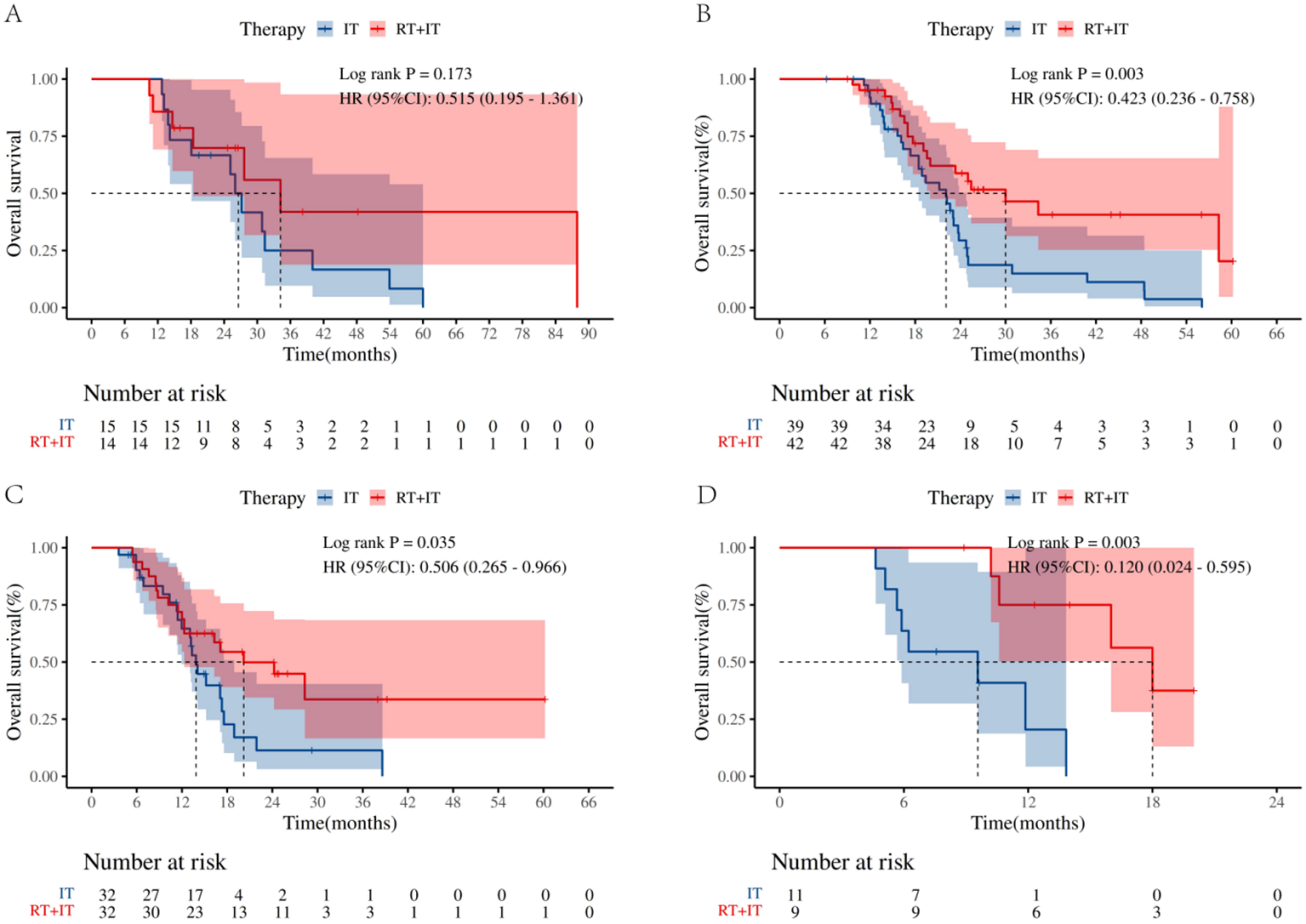
The subgroup of Kaplan–Meier survival curves comparing median OS of patients with PVTT I–IV who underwent RT+IT vs. IT after PSM: **(A)** PVTT I (median OS 34.20 vs. 26.58 months), **(B)** PVTT II (median OS 30.00 vs. 22.10 months) after PSM, **(C)** PVTT III (mOS 20.20 vs. 13.87 months), and **(D)** PVTT IV (median OS 18.00 vs. 9.57 months). OS, median overall survival; PSM, propensity score matching; RT, radiotherapy; IT, immunotherapy and targeted therapy; PVTT, portal vein tumor thrombus.

For patients with PVTT classification I, the mOS was 34.20 months (95% CI: 18.40 to not estimable) vs. 26.58 months (95% CI: 18.07 to not estimable) (HR: 0.515, 95% CI: 0.195–1.361, *p* = 0.173).
For patients with PVTT classification II, the mOS was 30.00 months (95% CI: 20.00 to not estimable) vs. 22.10 months (95% CI: 18.47–24.60) (HR: 0.423, 95% CI: 0.236–0.758, *p* = 0.003).
For patients with PVTT classification III, the mOS was 20.20 months (95% CI: 12.30 to not estimable) vs. 13.87 months (95% CI: 11.97–18.93) (HR: 0.506, 95% CI: 0.265–0.966, *p* = 0.035).
For patients with PVTT classification IV, the mOS was 18.00 months (95% CI: 16.00 to not estimable) vs. 9.57 months (95% CI: 5.89 to not estimable) (HR: 0.120, 95% CI: 0.024–0.595, *p* = 0.003) (**Figures 2A–D**).

### Progression-free survival analyses

After PSM, for PFS, there were 133 events in total, including 64/97 in the RT+IT group and 69/97 in the IT group. By PVTT subgroup, the PFS events were 7/14 vs. 10/15 for PVTT I, 30/42 vs. 24/39 for PVTT II, 22/32 vs. 25/32 for PVTT III, and 5/9 vs. 10/11 for PVTT IV (RT+IT vs. IT). HCC patients who received RT+IT had an mPFS of 12.30 months (95% CI: 10.47–16.45) vs. 7.20 months (95% CI: 6.37–9.67) (HR: 0.458, 95% CI: 0.319–0.656, *p* < 0.001) compared to those who received IT (**Figure 1B**).

**Figure 3:**
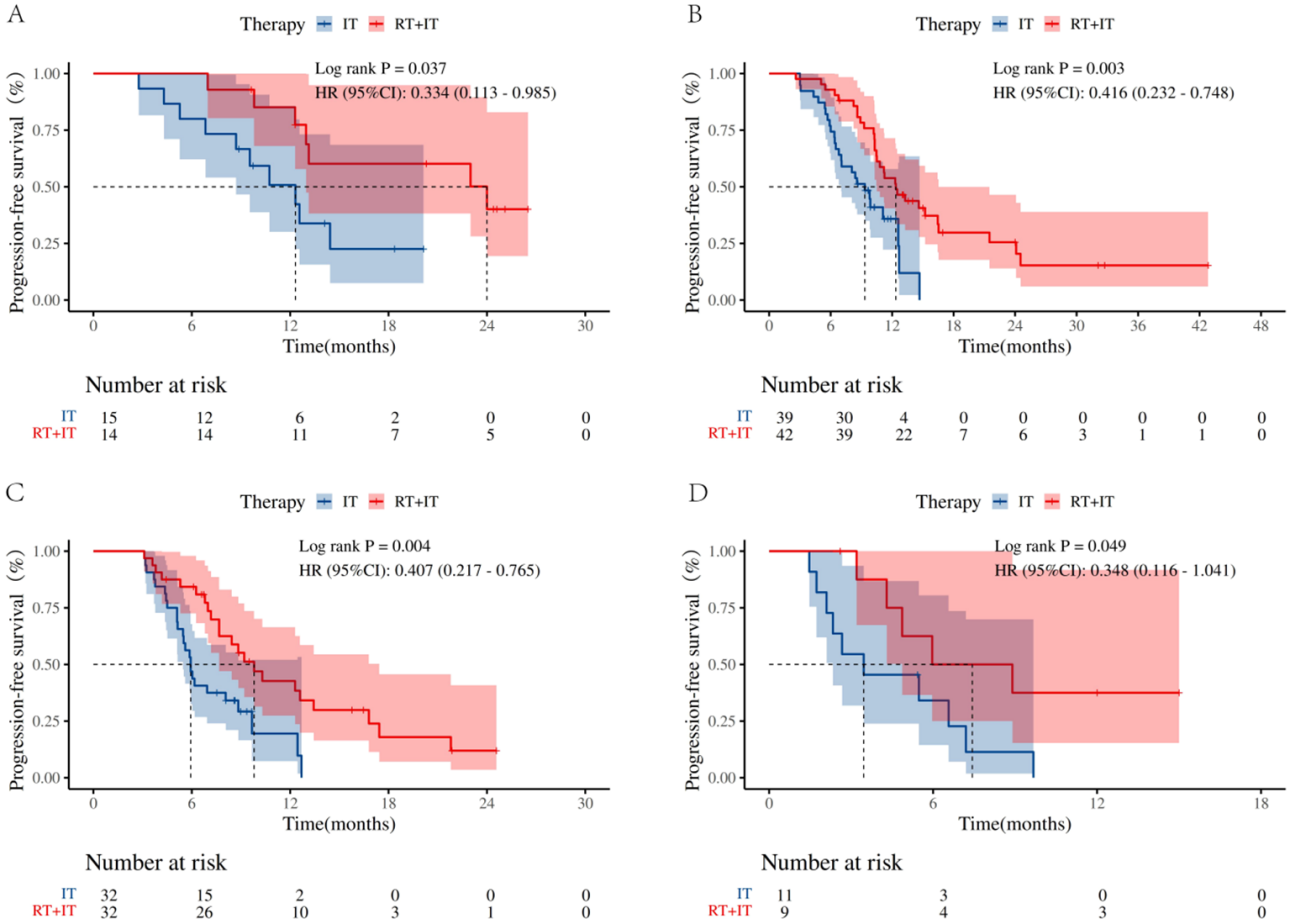
The subgroup of Kaplan–Meier survival curves comparing median PFS of patients with PVTT I–IV who underwent RT+IT vs. IT after PSM: **(A)** PVTT I (median PFS 24.00 vs. 12.32 months), **(B)** PVTT II (median PFS 12.36 vs. 9.33 months), **(C)** PVTT III (median PFS 9.80 vs. 5.93 months), and **(D)** PVTT IV (median PFS 7.43 vs. 3.46 months). PFS, progression-free survival; PSM, propensity score matching; RT, radiotherapy; IT, immunotherapy and targeted therapy; PVTT, portal vein tumor thrombus.

For patients with PVTT classification I, the mPFS was 24.00 months (95% CI: 12.97 to not estimable) vs. 12.32 months (95% CI: 8.70 to not estimable) (HR: 0.334, 95% CI: 0.113–0.985, *p* = 0.037).
For patients with PVTT classification II, the mPFS was 12.36 months (95% CI: 10.50–21.50) vs. 9.33 months (95% CI: 7.03 to not estimable) (HR: 0.416, 95% CI: 0.232–0.748, *p* = 0.003).
For patients with PVTT classification III, the mPFS was 9.80 months (95% CI: 7.67–17.43) vs. 5.93 months (95% CI: 5.47 to not estimable) (HR: 0.407, 95% CI: 0.217–0.765, *p* = 0.004).
For patients with PVTT classification IV, the mPFS was 7.43 months (95% CI: 4.87 to not estimable) vs. 3.46 months (95% CI: 2.33 to not estimable) (HR: 0.348, 95% CI: 0.116–1.041, *p* = 0.049) (**Figures 3A–D**).

### Treatment safety

Among the 312 patients before PSM, the incidence rates of leukopenia (32.39% vs. 16.91%, *p* = 0.002), increased AST (63.64% vs. 40.00%, *p* < 0.001), increased ALT (43.18% vs. 27.94%, *p* = 0.006), increased ALP (43.75% vs. 27.94%, *p* = 0.004), and decreased albumin (81.25% vs. 55.88%, *p* < 0.001) were higher in the RT+IT group (**Table 2**). Nonetheless, there was no difference in the frequency of grade 3–4 adverse events between the two groups. Among the 194 patients in the matched cohorts after PSM, in the RT+IT group, the frequency of leukopenia (32.99% vs. 14.43%, *p* = 0.002), increased AST (64.95% vs. 38.54%, *p* < 0.001), increased ALT (45.36% vs. 23.71%, *p* = 0.002), increased ALP (44.33% vs. 26.80%, *p* = 0.011), and decreased albumin (80.41% vs. 56.70%, *p* < 0.001) was higher than in the IT group. In the same way, there was no difference in the incidence of grade 3–4 adverse events between the matched cohorts. The percentage of cases of grade 3–4 adverse events did not differ throughout the matched cohorts.

**Table 2:** Treatment-related adverse events.

	Before PSM						After PSM					
Adverse event	Any grade			Grade 3–4			Any grade			Grade 3–4		
	RT+IT, *n* = 176 (%)	IT, *n* = 136 (%)	*p*	RT+IT, *n* = 176 (%)	IT, *n* = 136 (%)	*p*	RT+IT, *n* = 97 (%)	IT, *n* = 97 (%)	*p*	RT+IT, *n* = 97 (%)	IT, *n* = 97 (%)	*p*
Leukopenia	57 (32.39)	23 (16.91)	0.002	12 (6.82)	6 (4.41)	0.366	32 (32.99)	14 (14.43)	0.002	7 (7.22)	2 (2.06)	0.172
Thrombocytopenia	59 (33.52)	36 (26.47)	0.180	10 (5.68)	10 (7.35)	0.550	34 (35.05)	23 (23.71)	0.083	5 (5.15)	5 (5.15)	1.000
Gastrointestinal bleeding	7 (3.98)	3 (2.21)	0.578	2 (1.14)	2 (1.47)	1.000	2 (2.06)	1 (1.03)	1.000	0 (0.00)	2 (2.06)	0.477
Rash	22 (12.50)	14 (10.29)	0.545	3 (1.70)	4 (2.94)	0.729	13 (13.40)	14 (14.43)	0.836	3 (3.09)	3 (3.09)	1.000
Abdominal pain	29 (16.48)	24 (17.65)	0.785	9 (5.11)	4 (2.94)	0.341	17 (17.53)	15 (15.46)	0.699	4 (4.12)	1 (1.03)	0.365
Increased AST	112 (63.64)	54 (40.00)	<0.001	17 (9.66)	7 (5.15)	0.138	63 (64.95)	37 (38.54)	<0.001	13 (13.40)	7 (7.22)	0.157
Increased ALT	76 (43.18)	38 (27.94)	0.006	7 (3.98)	5 (3.68)	0.891	44 (45.36)	23 (23.71)	0.002	6 (6.19)	3 (3.09)	0.495
Increased ALP	77 (43.75)	38 (27.94)	0.004	0 (0.00)	0 (0.00)	1.000	43 (44.33)	26 (26.80)	0.011	0 (0.00)	0 (0.00)	1.000
Increased total bilirubin	102 (57.95)	65 (47.79)	0.074	13 (7.39)	7 (5.15)	0.423	58 (59.79)	45 (46.39)	0.061	7 (7.22)	5 (5.15)	0.551
Decreased albumin	143 (81.25)	76 (55.88)	<0.001	0 (0.00)	0 (0.00)	1.000	78 (80.41)	55 (56.70)	<0.001	0 (0.00)	0 (0.00)	1.000

Values are presented as *n* (%) and *p*-values were calculated using a two-sided chi-square test.

AST, aspartate aminotransferase; ALT, alanine aminotransferase; ALP, alkaline phosphatase; PSM, propensity score matching; IT, immunotherapy and targeted therapy; RT, radiotherapy.

### Multivariate Cox regression analysis for OS in the RT+IT combination group

Among the patients in the RT+IT combination treatment group, BED10, AFP, CP grade, Cheng’s classification of PVTT, and combined TACE were independent prognostic factors through multivariate Cox regression analysis (**Supplementary Table 1**). Assessment of the proportional hazards assumption suggested possible non-proportionality for several covariates included in the multivariate Cox regression analysis (**Supplementary Table 2**).

## Discussion

In contrast to immunotherapy combined with targeted therapy, our study demonstrated that IMRT combined with immunotherapy and targeted therapy was associated with improved OS and PFS in HCC patients with PVTT classification I–IV. Furthermore, the incidence of grade 3–4 toxicities did not significantly increase with the combined treatment. These findings highlight the encouraging effectiveness and tolerable security of IMRT in combination with immunotherapy and targeted therapy for HCC patients with PVTT I–IV. BED10, AFP, CP grade, and combined TACE independently predicted OS in patients receiving IMRT combined with immunotherapy and targeted therapy.

At present, the effectiveness and safety of RT with immunotherapy and targeted therapy in HCC patients with PVTT have been documented in certain studies, but there has been limited research focused on each classification of PVTT, especially Cheng’s classification of PVTT IV (23–26). Ma et al. recently reported that RT combined with TKI and PD-1 inhibitor therapy significantly prolonged survival compared with TKI plus PD-1 inhibitor alone in a propensity score-matched cohort, with an mOS of 15.6 months and an mPFS of 8.1 months in the RT-containing group (19). In a multicenter single-arm phase II study, Mo et al. evaluated SBRT combined with cadonilimab and lenvatinib in patients with Vp3/Vp4 PVTT and reported encouraging efficacy, including a primary lesion ORR of 38.1%–47.6%, a PVTT ORR of 76.2%, a median PFS of 6.8 months, and a median OS of 13.4 months (20). Their results are consistent with our conclusion that incorporation of RT may be associated with survival benefit in patients with PVTT. However, our study extends that work in several respects. First, our cohort was larger, including 312 patients overall and 97 matched pairs after PSM. Second, we focused specifically on IMRT-based treatment and included patients across PVTT I–IV, allowing a broader evaluation across Cheng’s classification. Additionally, the prospective trial of Mo et al. strengthens the rationale for combining RT with systemic therapy in advanced PVTT. However, we included PVTT I–IV rather than only Vp3/Vp4 disease, used a comparative design with an IT-alone control group, and reflected real-world IMRT-based practice across multiple immune-targeted regimens. Therefore, our data complement the prospective single-arm evidence by providing comparative real-world evidence on the incremental benefit of adding RT to immune-targeted therapy.

Our earlier research on HCC patients with PVTT revealed no discernible difference between IMRT and SBRT in terms of local control rate, OS, or PFS (10). Additionally, IMRT plus anti-PD-1 did not raise the incidence of radiation-induced liver toxicity compared with IMRT alone (27). The interaction between radiation technique and systemic therapy deserves further discussion. SBRT delivers a high biologically effective dose in a short overall treatment time and may generate more pronounced immunogenic cell death and tumor-antigen release, which provides a biologic rationale for combination with ICIs. Conversely, IMRT may be preferred for patients with large intrahepatic tumors, extensive PVTT, or tumors close to the stomach or duodenum, because its more fractionated schedule can improve target coverage and OARs sparing while preserving liver reserve. Therefore, the benefit of adding RT to IT should not be interpreted solely as local cytoreduction of PVTT; improved control of macrovascular invasion may also maintain portal venous flow, reduce portal hypertension-related deterioration, preserve hepatic function, and thereby help patients continue and benefit from systemic therapy. Prospective data supporting this concept include the multicenter study of IMRT combined with atezolizumab and bevacizumab, which showed an ORR of 76.6%, a median OS of 9.8 months, and a median PFS of 8.0 months in HCC with extrahepatic PVTT (28). Furthermore, the ongoing phase III NRG-GI012 (HELIO-RT; NCT07166406) trial is randomizing patients with HCC and macrovascular invasion to immunotherapy-based systemic therapy alone vs. immunotherapy-based systemic therapy plus liver SBRT and was available for patient enrollment now. The results of this randomized trial are expected to define more rigorously whether the addition of SBRT improves survival beyond systemic therapy alone in this population.

The prognosis for patients with HCC who have PVTT is poor, and the effectiveness of treatment is made more difficult by the existence of important risk factors (2, 4, 29). In patients with HCC, PVTT incidence varies between 44% and 62.2% (30), which is categorized as advanced stage, and is typically associated with fewer curative options (31). The IMbrave150 research demonstrated the critical role of immunotherapy plus targeted therapy (7). According to Ning et al., patients who received RT combined with immune-targeted triple therapy achieved better disease control rate (100% vs. 75.9%, *p* = 0.005) compared to immune-targeted therapy alone, without significantly increasing toxicity and side effect (18). These results demonstrated that RT combined with immunotherapy and targeted therapy is an efficient and secure treatment approach for advanced HCC patients. Despite these promising results, limited research has explicitly examined the application of RT combined with immunotherapy and targeted therapy for HCC patients with PVTT.

The mOS of RT combined with sintilimab and bevacizumab for the initial therapy of HCC having PVTT was 24.0 months (95% CI: 19.0 to not applicable) in a recent phase II single-arm research consisting of 46 HCC patients having PVTT (32). Additionally, Hu et al. conducted a prospective randomized clinical study consisting of HCC patients having PVTT. The study showed a significantly higher 12-month OS rate in the SBRT plus camrelizumab and apatinib group than in the camrelizumab and apatinib group (51.3% vs. 23.1%) (33). These results all confirm the effectiveness of RT combined with immunotherapy and targeted therapy in HCC patients with PVTT. Nevertheless, in the IT group, all patients showed disease progression at 24 months; regarding the poorer long-term PFS in the IT group, this is likely related to the aggressive nature of HCC with PVTT, which is well known to be associated with rapid disease progression, impaired portal venous flow, deterioration of liver function, and limited treatment responsiveness. The late PFS estimates, especially in the IT group, are based on a limited number of patients remaining at risk. Importantly, this issue does not alter the main conclusion of our study, but the Kaplan–Meier estimates, particularly long-term PFS, become less stable and should therefore be interpreted with caution.

Our analysis revealed that for HCC patients having PVTT who received RT with immunotherapy and targeted therapy, BED10, AFP, CP grade, Cheng’s classification of PVTT, and combined TACE were independently associated with OS. In addition, some covariates did not fully satisfy the proportional hazards assumption. Therefore, the HRs derived from the Cox regression should be interpreted with caution, as they may reflect average effects over the follow-up period rather than constant effects over time. Although this does not change the overall direction of our findings, it represents a limitation of the Cox-based analysis. BED10 >75 Gy extended OS for HCC patients with PVTT, according to one study (34). According to a different study, HCC patients with PVTT undergoing SBRT had higher OS if their BED10 was greater than 100 Gy (10). The standard course of treatment for unresectable HCC with PVTT I–III is RT with TACE (11, 12). The efficacy of PVTT III–IV remains far from satisfactory. A Japanese study showed that when the PVTT extends beyond the first-order branch, the postoperative recurrence-free survival was only 0.38 years, resulting in the advice against liver resection for PVTT III/IV patients (35). Furthermore, when the primary portal vein is totally blocked, the applicability of TACE is significantly restricted (36). Prior TACE combined with RT and immuno-targeted therapy had a better prognosis, according to C regression analysis. This suggests that the efficacy of TACE combined with RT and immuno-targeted therapy may be better than RT with immunotherapy and targeted therapy for HCC patients having PVTT. However, large-scale population studies must be performed to validate these results.

Being a low-toxicity and exceptionally effective medication, RT plays a critical role in managing PVTT classification III–IV. Compared to sorafenib, in patients with PVTT classification IV, RT has been demonstrated to enhance OS (37). In our investigation, patients with PVTT classification III who received RT plus immunotherapy and targeted therapy had an mOS of 28.30 months, and for PVTT classification IV, it was 18.00 months, substantially exceeding previously reported outcomes (10.9 months). PD-1 inhibitors and RT may have a synergistic therapeutic benefit. Studies have demonstrated that RT for HCC can modify the tumor immune microenvironment, via increasing programmed cell death protein ligand 1 (PD-L1), which suppresses CD8^+^ T-cell activity and promotes tumor cells’ immunological escape. The immunosuppressive state caused by RT can be counteracted by PD-1 inhibitors and can also cause the regression of tumors outside the RT area, resulting in distal effects (15, 38). Additionally, TKIs and vascular endothelial growth factor (VEGF) inhibitors further enhance immunity and sensitize tumors to checkpoint blockade therapy (39).

The timing of immunotherapy and targeted therapy relative to RT is clinically important because conventional or hypofractionated IMRT often requires a longer treatment course than SBRT, increasing the potential overlap with systemic agents. In the phase II study of sintilimab plus bevacizumab combined with IMRT, RT was initiated after two cycles of systemic therapy, and systemic therapy was withheld during IMRT and resumed 2 weeks after RT (32). A recent narrative review also emphasized that no universally accepted guideline exists for combining RT with systemic targeted or immune therapies in HCC; available data suggest that ICIs combined with RT are generally tolerable, whereas sorafenib and bevacizumab require greater caution because of potential hepatotoxicity and gastrointestinal toxicity (40). In the present real-world cohort, we therefore used a pragmatic 4-week window to define concurrent or sequential IMRT combined with immunotherapy and targeted therapy and allowed physician-directed interruption of targeted agents when safety concerns arose. Interruptions in the administration of immunotherapy and targeted agents occurred in a small proportion of patients, which may have influenced clinical outcomes. Because the timing and duration of drug holding were not prospectively standardized, the survival benefit observed with IMRT combined with immunotherapy and targeted therapy should be interpreted as reflecting the overall multimodal treatment strategy rather than a specific optimal sequence or drug-interruption policy.

There were certain restrictions on our investigation. Initially, this was a retrospective, single-center trial, and there may have been selection bias. Secondly, the RT+IT group included multiple immune checkpoint inhibitors, targeted agents, radiation dose schedules, and sequencing strategies. Although our results should be interpreted as reflecting the overall effectiveness of this combined treatment approach rather than the efficacy of any specific regimen, further prospective studies are needed to define the optimal combination strategy. Lastly, extending the follow-up period may facilitate a better assessment of the benefits of RT combination therapy on survival outcomes.

## Conclusion

In conclusion, our study showed that IMRT combined with immunotherapy and targeted therapy was associated with improved survival outcomes in HCC patients with PVTT classification I–IV. The probability of grade 3–4 treatment-related side events was comparable to that in patients receiving immunotherapy and targeted therapy.

## Data Availability

The raw data supporting the conclusions of this article will be made available by the authors, without undue reservation.
