# Supplementary material for: Survival outcomes of intensity-modulated radiotherapy combined with immunotherapy and targeted therapy for hepatocellular carcinoma with classification I–IV portal vein tumor thrombus: a propensity score matching study
Source: Front Immunol. 2026 Jul 28;17:1775165. doi: 10.3389/fimmu.2026.1775165 (PMC13457297; doi:10.3389/fimmu.2026.1775165)
Supplement: Supplementary file 1 [file Table1.docx]

Supplement Table 1**.** Univariate and multivariate analysis of overall survival in the RT+IT combination group before PSM.

| Variables | Univariate analysis | | Multivariate analysis | | | |
| --- | --- | --- | --- | --- | --- | --- |
|  | HR (95%CI) | *P* | HR (95%CI) | *P* | | |
| Age(years) | 0.99 (0.97 ~ 1.01) | 0.454 |  |  | | |
| Sex,male vs. female | 2.12 (0.67 ~ 6.73) | 0.201 |  |  | | |
| Hepatitis B virus infection,  positive vs.negative | 0.85 (0.52 ~ 1.38) | 0.508 |  |  | | |
| Cirrhosis,present vs.absent | 1.15 (0.74 ~ 1.79) | 0.548 |  |  | | |
| ECOG PS,1 vs. 0 | 1.27 (0.82 ~ 1.96) | 0.288 |  |  | | |
| Tumor number,>3 vs. ≤4 | 1.45 (0.94 ~ 2.25) | 0.096 |  |  | |  |
| Max tumor size(cm) | 1.04 (0.99 ~ 1.10) | 0.126 |  |  |  |  |
| Alpha fetoprotein(ng/ml),≥400 vs. <400 | 2.51 (1.60 ~ 3.93) | <0.001 | 1.88 (1.15 ~ 3.06) | 0.011 |  |  |
| Child-Pugh grade,B vs. A | 1.90 (1.17 ~ 3.10) | 0.010 | 1.78 (1.05 ~ 3.02) | 0.032 | |  |
| ALBI grade 2 vs.1 | 1.68 (0.80 ~ 3.50) | 0.169 |  |  | |  |
| Aspartate aminotransferase(U/L) | 1.01 (1.01 ~ 1.01) | 0.031 |  |  | |  |
| Alanine aminotransferase(U/L) | 1.00 (1.00 ~ 1.00) | 0.072 |  |  | |  |
| Alkaline phosphatase(U/L) | 1.00 (1.00 ~ 1.00) | 0.162 |  |  | |  |
| Prothrombin time (sec) | 1.19 (1.03 ~ 1.38) | 0.019 |  |  | |  |
| Cheng’s classification of PVTT  II vs.I | 1.22 (0.63 ~ 2.35) | 0.549 | 0.89 (0.45 ~ 1.74) | 0.725 | |  |
| III vs.I | 2.08 (1.06 ~ 4.08) | 0.033 | 1.49 (0.73 ~ 3.03) | 0.273 | |  |
| IV vs.I | 2.79 (1.20 ~ 6.49) | 0.017 | 1.15 (0.45 ~ 2.95) | 0.769 | |  |
| Combined TACE,Yes vs.No | 0.58 (0.36 ~ 0.94) | 0.028 | 0.51 (0.31 ~ 0.85) | 0.009 | |  |
| BED_10_(Gy) | 0.95 (0.93 ~ 0.97) | <0.001 | 0.96 (0.93 ~ 0.99) | 0.005 | |  |

ALBI, albumin-bilirubin; BED_10_, biologically effective dose (calculated using LQ model, α/β=10); ECOG PS, Eastern Cooperative Oncology Group-performance status; IT, immunotherapy and targeted therapy; PSM, propensity score matching; PVTT, portal vein tumor thrombus; RT, radiotherapy; TACE, transcatheter chemoembolization; HR, hazard ratio; CI, Confidence Interval.
