# Supplementary material for: Survival outcomes of intensity-modulated radiotherapy combined with immunotherapy and targeted therapy for hepatocellular carcinoma with classification I–IV portal vein tumor thrombus: a propensity score matching study
Source: Front Immunol. 2026 Jul 28;17:1775165. doi: 10.3389/fimmu.2026.1775165 (PMC13457297; doi:10.3389/fimmu.2026.1775165)
Supplement: Supplementary file 2 [file Table2.docx]

Supplement Table 2**.** Proportional hazards assumption for the Cox regression

| Variable | Coefficient (B) | SE | HR | P value | Interpretation |
| --- | --- | --- | --- | --- | --- |
| AFP (≥400 vs. <400) | -0.185 | 0.033 | 0.831 | <0.001 | Possible violation of PH assumption |
| Child-Pugh grade (B vs. A) | -0.294 | 0.066 | 0.745 | <0.001 | Possible violation of PH assumption |
| Combined TACE (Yes vs. No) | -0.169 | 0.023 | 0.845 | <0.001 | Possible violation of PH assumption |
| BED10 | -0.014 | 0.001 | 0.986 | <0.001 | Possible violation of PH assumption |
| Cheng’s classification of PVTT (II vs. I) | 0.001 | 0.005 | 0.999 | 0.88  4 | No evidence of PH assumption violation |
| Cheng’s classification of PVTT (III vs. I) | 0.010 | 0.006 | 1.010 | 0.107 | No evidence of PH assumption violation |
| Cheng’s classification of PVTT (IV vs. I) | -0.172 | 0.064 | 0.842 | 0.007 | Possible violation of PH assumption |

AFP,alpha fetoprotein;BED10,biologically effective dose (calculated using LQ model, α/β=10);HR,hazard ratio;PH,proportional hazards;IT, immunotherapy and targeted therapy;PSM, propensity score matching;PVTT,portal vein tumor thrombus;SE,

standard error;RT, radiotherapy;TACE,transcatheter chemoembolization
